# Renal replacement therapy in very elderly critical care patients

**DOI:** 10.1186/cc13582

**Published:** 2014-03-17

**Authors:** K Brown, HG Jones

**Affiliations:** 1 Morriston Hospital, Swansea, UK

## Introduction

The very elderly (>80 years) UK population is increasing and along with it there is an increased utilisation of critical care resources [1]. The use of chronological age as a bar to treatment is unethical, yet a discrepancy of opinion remains between the disinclined and the elderly advocate intensivist [2]. Acute kidney injury requiring dialysis is a frequent complication of critical illness and is associated with high morbidity and mortality. An increased risk is seen with age due to the high prevalence of risk factors and chronic kidney disease in the elderly [3].

## Methods

Very elderly patients admitted to our tertiary referral ITU were included in a 2-year (December 2011 to November 2013) retrospective cohort analysis. The outcome of those receiving RRT was reviewed.

## Results

There were 2,297 admissions to the ITU, of which 323 (14%) were classified as very elderly with a mean APACHE II score of 17.3. RRT was utilised in 12.4% (*n *= 40) of the very elderly patients with a mean APACHE II score of 25. Forty-five per cent of AKI was due to sepsis and 25% due to emergency surgery. ITU very elderly patient mortality was 30.1%, and in those who received RRT was 42% and 67% at ITU and hospital discharge respectively. Recovery of renal function was seen in 19 patients at ITU discharge, of these 11 survived to hospital discharge. Two patients required ongoing IHD in the community.

## Conclusion

Current predictions estimate that the very elderly UK population will almost double by 2030 [4]. The biggest uptake in acceptance of chronic RRT has been in the elderly [5], where no difference has been shown in health-related quality of life from the general dialysis population [6]. Our experience suggests that RRT has a role in the critically ill elderly patient, with 33% surviving to hospital discharge. The challenge is identifying those most likely to benefit from a cohort with multiple comorbidities, against the available resources, rising demand for critical care in an aging population and increasing expectations [7].
